# Quantitative comparison of mean macular thickness in COVID-19 patients versus healthy individuals using optical coherence tomography

**DOI:** 10.1186/s12886-025-04177-7

**Published:** 2025-08-11

**Authors:** Nagilton Bou Ghosn, Marcos Balbino, Ana Carolina Pasquini Raiza, Camila Sayuri Vicentini Otani, Victoria Moreira Fernandes, Vitor Kazuo Lotto Takahashi, Beatriz Silveira Seixas, Laura Vanalli Guimarães, Luiz Paulo Dias Mario, Regina Cele Silveira Seixas

**Affiliations:** 1 Grupo OPTY - HCLOE Oftalmologia Especializada, São Paulo, Brazil; 2 Centro Universitário São Camilo, Rua Cipriano Barata, 1869– ap. 93, São Paulo, SP Brazil; 3 Medical Sciences of Minas Gerais Eye Institute (IOCM-MG), Belo Horizonte, MG Brazil; 4 Universidade de Santo Amaro, UNISA, São Paulo, Brazil

**Keywords:** COVID-19, Optical coherence tomography, Macular thickness, Retinal imaging, Age stratification, OCTA, Retina, SARS-CoV-2, Vascular retina changes, Retinal inflammation

## Abstract

**Background:**

SARS-CoV-2 is associated with systemic inflammation, vascular dysfunction, and potential ocular involvement. While structural retinal changes have been observed in some patients, the long-term impact of COVID-19 on macular architecture remains unclear, §particularly in unvaccinated populations.

**Objective:**

To evaluate differences in central macular thickness (CMT) between post-COVID-19 patients and healthy individuals using optical coherence tomography (OCT), with age-stratified analysis.

**Design:**

A prospective case-control study conducted at a specialized ophthalmology center in Brazil during the early vaccination phase. A total of 76 unvaccinated participants were included: 29 patients with prior COVID-19 (58 eyes) and 47 healthy controls (94 eyes). OCT was performed at least 14 days after PCR-confirmed infection.

**Results:**

The overall mean CMT was 246.93 ± 23.30 μm. No significant difference was found between the COVID-19 and control groups (242.54 ± 19.76 μm vs. 249.63 ± 25.06 μm; *p* = 0.10). However, among participants aged ≥ 42 years, post-COVID-19 patients had significantly lower CMT compared to age-matched controls (241.00 ± 21.90 μm vs. 256.85 ± 28.58 μm; *p* = 0.04). No significant difference was observed in the < 42 years group. OCT angiography revealed no qualitative vascular abnormalities in either group.

**Conclusion:**

Our findings suggest that SARS-CoV-2 infection may be associated with subtle retinal thinning in older adults, even in the absence of acute symptoms or visual complaints. These results highlight the importance of age-stratified analysis and support further investigation into the long-term ocular effects of COVID-19.

## Introduction

Since its emergence in December 2019, SARS-CoV-2 has led to a global health crisis, with widespread systemic effects that extend beyond the respiratory system. While initial research focused primarily on pulmonary complications, growing evidence suggests that the virus may also affect the cardiovascular, neurological, and ocular systems. However, the ophthalmologic manifestations of COVID-19 remain incompletely understood [1, 2]. 

Several systemic diseases, such as diabetes mellitus, systemic arterial hypertension, tuberculosis, and systemic lupus erythematosus are known to affect ocular structures, including the retina and optic nerve [1]. Animal studies have demonstrated that coronaviruses can induce ocular conditions such as conjunctivitis, anterior uveitis, retinitis, and optic neuritis in feline and murine models [2]. These findings raise important concerns about the potential impact of SARS-CoV-2 on the human visual system.

One of the hallmark complications of COVID-19 is the promotion of a hypercoagulable and proinflammatory state, leading to endothelial dysfunction, vascular occlusion, and ischemia [3]. Given the shared embryological origin and vascular supply between the retina and the brain, the retina may serve as a useful site for detecting subtle systemic vascular injury. Indeed, previous studies have reported findings such as cotton wool spots, hemorrhages, and increased vascular tortuosity in COVID-19 patients [4–6, 7]. 

Optical coherence tomography (OCT) is a non-invasive imaging technique that enables high-resolution cross-sectional visualization of retinal structures. It is widely used in ophthalmology and has proven valuable in detecting retinal alterations in both ocular and systemic diseases [5–8]. Despite its utility, the use of OCT in evaluating retinal changes after SARS-CoV-2 infection has been limited, especially in patients who were not hospitalized or vaccinated [1–3]. 

Although some studies have reported increased or unchanged retinal thickness following COVID-19, most of these analyses did not include age stratification, nor did they focus on unvaccinated individuals [4, 9]. Moreover, discrepancies remain in the literature, with some authors reporting structural or vascular abnormalities and others finding no significant differences [4, 7, 10].

Given these inconsistencies, the present study aimed to quantitatively and qualitatively assess retinal changes in patients with a recent history of PCR-confirmed COVID-19 using spectral-domain OCT (SD-OCT). Specifically, we evaluated central macular thickness (CMT), the presence of hyperreflective lesions, and peripapillary retinal nerve fiber layer (pRNFL) changes, and compared these parameters with a healthy control group.

## Methods

This prospective case-control study was conducted in São Paulo, Brazil, at HCLOE– Clínica de Oftalmologia Especializada, part of the OPTY Group. The study was conducted in accordance with the Declaration of Helsinki and approved by the institutional ethics committee. Written informed consent was obtained from all participants (Annex 1).

The COVID-19 group included 29 patients with confirmed SARS-CoV-2 infection based on RT-PCR (58 eyes), with data collected between August and December 2021. All patients were ≥ 18 years old, experienced mild to moderate symptoms, did not require hospitalization, and had their ophthalmological evaluation at least 14 days after diagnosis (range: 14–70 days post-infection). None of the COVID-19 patients had received any dose of COVID-19 vaccine at the time of OCT. The control group consisted of 47 healthy individuals with no history of SARS-CoV-2 infection (94 eyes); 38.3% of controls had received one vaccine dose.

All participants underwent a complete ophthalmological examination performed by retina specialists to rule out retinal or optic nerve pathology. Visual acuity was assessed using Snellen charts. CMT measurements were obtained via the device’s automated segmentation algorithm and reviewed by retina specialists. Posterior pole images were acquired using a swept-source OCT (DRI OCT Triton, Topcon Inc., Tokyo, Japan), with wavelength of 1050 nm and acquisition speed of 100,000 A-scans/sec. Each volumetric scan was 33 mm, composed of 320 A-scans per B-scan and 320 B-scans.

Exclusion criteria included diabetes, smoking, anticoagulant use, high myopia, ocular surgeries or pathologies, systemic vascular diseases, and eyes with significant refractive error (greater than ± 3.0 diopters) were excluded.

Automated segmentation of the superficial and deep capillary plexuses (SCP and DCP) was performed using built-in software (IMAGEnet6, v1.23.15008), and manually reviewed by masked retina specialists. Central macular thickness (CMT) was derived from the software’s automated measurements, with manual corrections when necessary.

## Results

A total of 76 patients (152 eyes) were enrolled: 29 post-COVID-19 individuals (58 eyes) and 47 controls (94 eyes). Data collection occurred between August and December 2021, during the early phase of COVID-19 vaccination in Brazil. All COVID-19 cases were confirmed by RT-PCR at least 14 days before examination, with no patients requiring hospitalization.

The cohort comprised 51 women (67.1%) and 25 men (32.9%). The COVID-19 group included a significantly higher proportion of females (84.0%) compared to the control group (50.98%) (*p* = 0.005). The mean age was 42.79 ± 17.20 years. No participant in the COVID-19 group had received any dose of COVID-19 vaccine; in contrast, 38.3% of controls had received at least one dose. Detailed demographic characteristics of the study groups are summarized in Table 1.

**Table 1:**
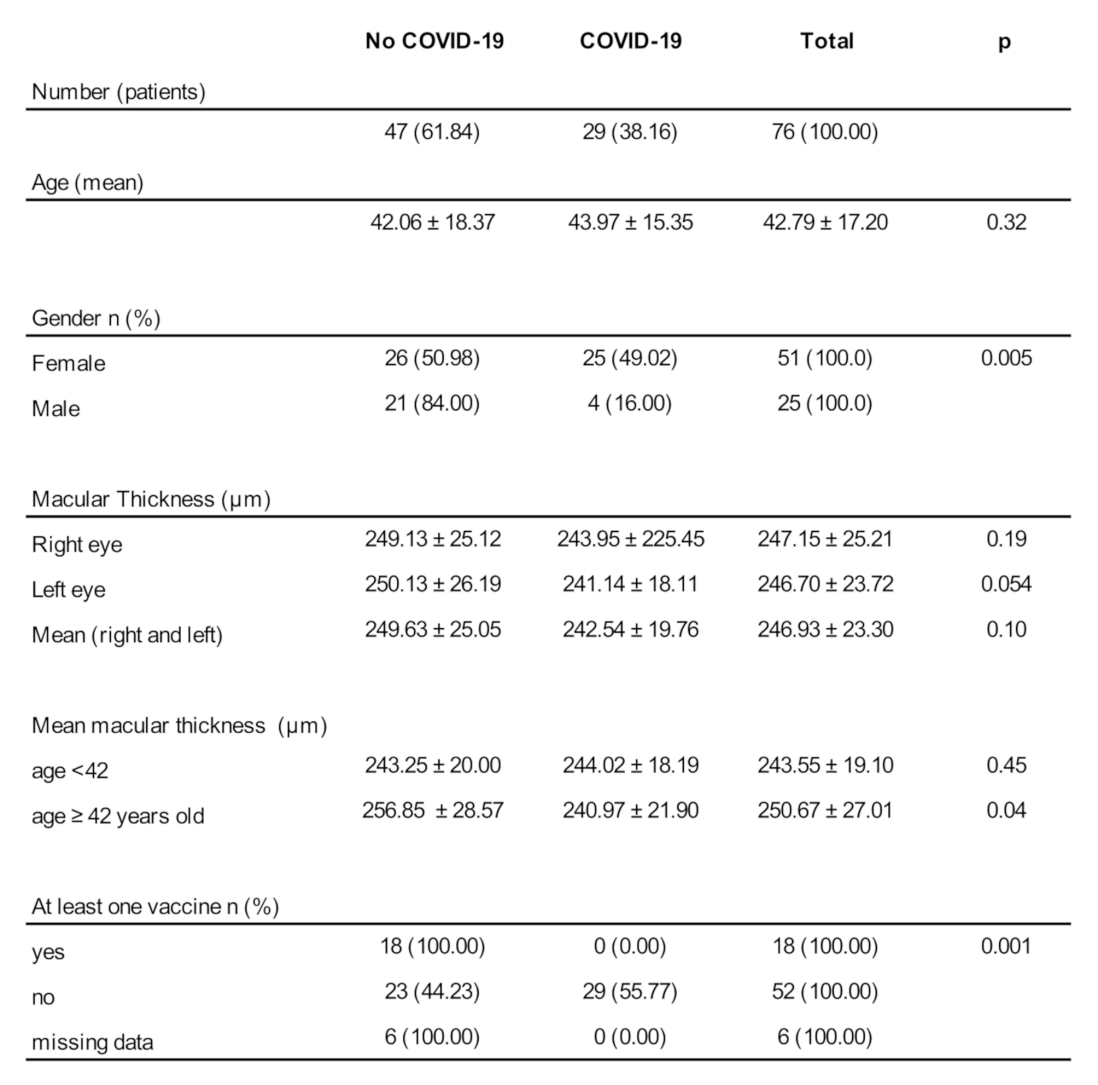
Demographic characteristics of the study participants. Summary of age, sex distribution, and vaccination status in the COVID-19 and control groups. Statistical comparisons were performed using t-tests or chi-square tests, with significance set at *p* < 0.05

**Fig. 1:**
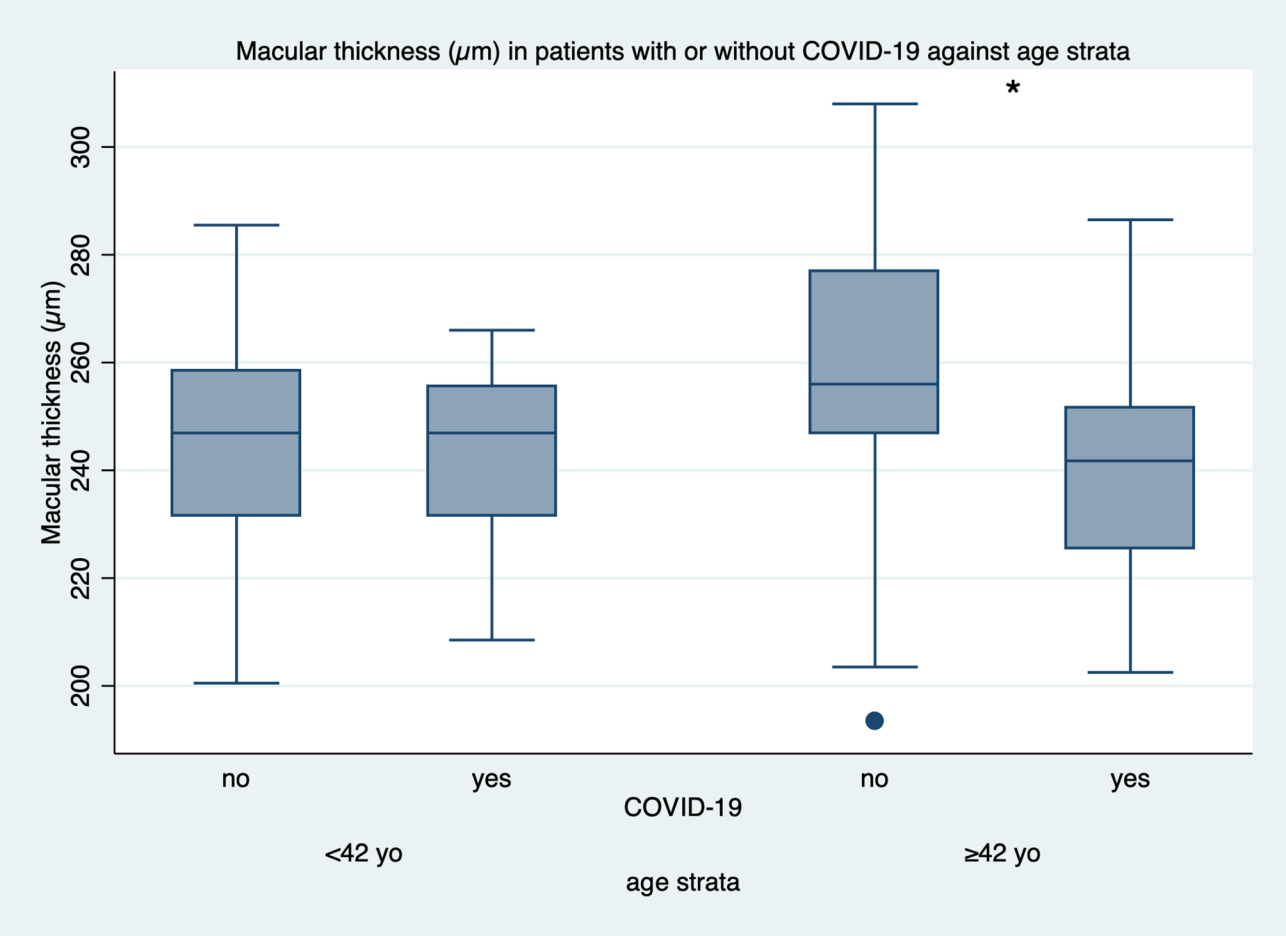
Mean central macular thickness in COVID-19 and control groups. Boxplot illustrating the distribution of central macular thickness (CMT) in the control and COVID-19 groups. No significant difference was found between the two groups (*p* = 0.10)

The overall mean central macular thickness (CMT) was 246.93 ± 23.30 μm. There was no statistically significant difference in CMT between the total COVID-19 and control groups (242.54 ± 19.76 μm vs. 249.63 ± 25.06 μm; *p* = 0.10), as illustrated in Fig. 1.

**Fig. 2:**
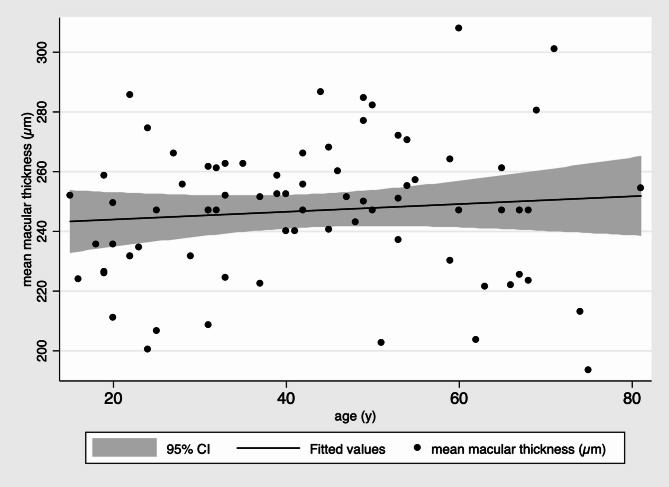
Central macular thickness in age-stratified groups. Bar graph comparing mean CMT in individuals younger than 42 years and 42 years or older. A significant reduction in CMT was observed in the older COVID-19 subgroup compared to controls (*p* = 0.04)

However, subgroup analysis by age revealed significant findings. In participants aged ≥ 42 years, COVID-19 patients exhibited a statistically significant reduction in CMT compared to controls (241.00 ± 21.90 μm vs. 256.85 ± 28.58 μm; *p* = 0.04). In contrast, no significant difference was observed in the < 42-year subgroup (*p* > 0.05). These results are displayed in Fig. 2. In contrast, no significant difference was found among participants younger than 42 years (*p* > 0.05).

**Fig. 3:**
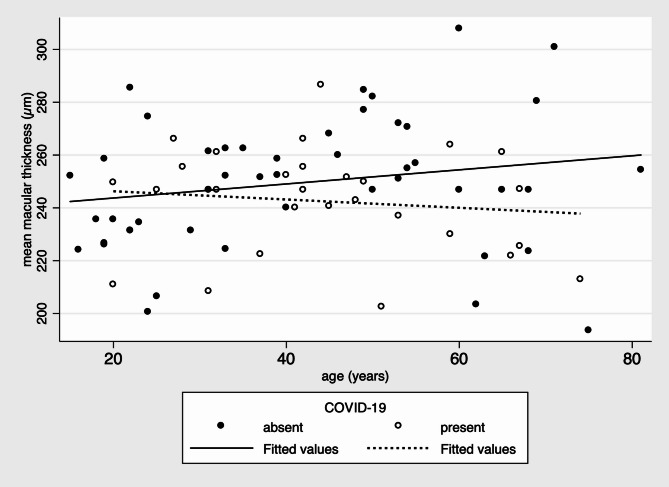
Correlation between age and central macular thickness. Scatter plot showing the correlation between age and CMT. A weak positive correlation was observed in the control group (*r* = 0.20), while a negative trend was seen in the COVID-19 group (*r*=-0.12)

Correlation analysis showed differing trends between groups. In the control group, there was a weak positive correlation between age and CMT (*r* = 0.20), suggesting a slight increase in macular thickness with age. Conversely, in the COVID-19 group, a weak negative correlation (*r*=–0.12) was noted, indicating a trend toward macular thinning with increasing age (Fig. 3). While not statistically significant, these patterns support the hypothesis of age-modulated post-infectious retinal effects.

Regarding OCT angiography, no structural abnormalities were detected in either group. Capillary plexuses were intact, and no signs of ischemia, non-perfusion zones, or neovascularization were identified. The automated segmentation was stable, and manual reviews by masked retina specialists confirmed the absence of qualitative alterations.

## Discussion

In this prospective case-control study, we investigated the potential effect of prior SARS-CoV-2 infection on central macular thickness in unvaccinated individuals using swept-source OCT. Our data showed a measurable reduction in central macular thickness (CMT) in patients with a history of COVID-19, particularly in those aged ≥ 42 years. While no significant differences were observed in the younger subgroup, older post-COVID-19 individuals exhibited significantly lower mean CMT compared to age-matched controls. This age-stratified difference suggests that retinal changes following COVID-19 may be modulated by age, potentially due to increased susceptibility to endothelial dysfunction, microvascular inflammation, or neuroretinal degeneration [3, 4, 7]. 

These findings contrast with prior studies that did not stratify by age. Oren et al. [9] for example, reported increased CMT and thinning of ganglion and inner nuclear layers in post-COVID-19 patients, but their evaluations were limited to 14–30 days post-infection and did not include stratification. Additionally, their cohort was not specifically unvaccinated, making direct comparison complex. Our results better reflect real-world outpatient follow-up during the early stages of the pandemic in Brazil, where vaccination was not yet broadly implemented [4]. 

Kal et al. [11] demonstrated retinal thinning associated with elevated IL-6 and D-dimer levels in acute COVID-19, reinforcing the role of systemic inflammation and thrombosis in retinal compromise. While we did not assess systemic biomarkers, our findings support the possibility of delayed or persistent retinal structural effects in older patients.

Other studies reported inconsistent findings. Abrishami et al. [4] found no quantitative differences in retinal thickness but described hyperreflective lesions, while Invernizzi et al. [7] documented microvascular changes including hemorrhages and vascular tortuosity. These discrepancies may reflect differences in methodology, timing of exams, vaccination status, and lack of stratification by age or severity.

In our cohort, the correlation between age and CMT diverged between groups: a weak positive association in controls contrasted with a negative trend in COVID-19 patients, suggesting an age-dependent post-infectious degenerative pattern. Previous studies in healthy populations have demonstrated that macular thickness decreases with age, [12, 13]. Most of our OCT exams were performed several weeks after infection (14–70 days), when acute-phase mediators were likely diminished. However, persistent or subclinical retinal remodeling may still occur.

Our findings align with reports of neuronal and glial vulnerability in the aging retina and raise the hypothesis that SARS-CoV-2 may trigger or accelerate neuroretinal loss, particularly in older adults. Spectral-domain OCT has demonstrated sensitivity in detecting such structural changes, even when functional vision remains unaffected. Notably, OCT angiography revealed no microvascular alterations, suggesting that structural thinning may precede vascular compromise or reflect neurodegenerative rather than ischemic mechanisms [3, 4, 7]. 

Limitations of our study include the absence of baseline pre-infection OCTs, unavailability of serum biomarkers (e.g., IL-6, TNF-α), and lack of a defined post-infection imaging window. Although the use of a fully unvaccinated cohort strengthens internal validity, the lack of longitudinal follow-up restricts conclusions on disease progression or recovery. The influence of outliers, particularly in the control group and may have also affected linear modeling, though data were carefully reviewed and corrected by masked retina specialists. Another important limitation is that the study was conducted before COVID-19 vaccination became widely available in Brazil, meaning that the observed changes represent the natural, unmodulated effects of infection.

To our knowledge, this is one of the few studies to evaluate macular structural changes post-COVID-19 in an unvaccinated population using swept-source OCT with masked analysis. The implementation of age stratification allowed us to uncover findings that would likely remain hidden in pooled comparisons. Additional longitudinal studies are warranted to assess the persistence or reversibility of these changes and to determine whether they carry functional consequences over time.

In conclusion, SARS-CoV-2 may have subtle but measurable effects on the macular architecture of older adults, even after mild disease. These changes, although not accompanied by OCTA abnormalities, may signal long-term consequences for retinal integrity. Further longitudinal studies are needed to evaluate the persistence and clinical relevance of such findings, ideally incorporating functional testing, systemic biomarkers, and multimodal retinal imaging.

## Conclusion

Our findings demonstrate a statistically significant reduction in central macular thickness (CMT) among post-COVID-19 patients aged ≥ 42 years compared to age-matched healthy controls. While overall CMT values were similar between the COVID-19 and control groups, age-stratified analysis revealed that older individuals recovering from COVID-19 may experience subtle retinal structural changes.

Although no microvascular alterations were detected on OCT angiography, the reduction in CMT observed in older patients raises concerns about potential long-term retinal effects of SARS-CoV-2 infection, particularly in unvaccinated individuals. As human coronaviruses have been shown to affect ocular tissues in both animal models and humans, this evidence supports further investigation into post-infectious retinal remodeling.

It is important to acknowledge the context in which this study was conducted: during the early phase of the COVID-19 vaccination rollout in Brazil, a middle-income country where scientific uncertainty prevailed and vaccine access was limited. At the time, little was known about the long-term systemic or ophthalmic consequences of the infection. Our study reflects this unique period of global vulnerability and medical ambiguity, in which even the possibility of retinal involvement remained speculative.

These findings underscore the need for continued research using larger cohorts, multimodal retinal imaging, functional testing, and systemic biomarker analysis to better characterize the ocular implications of COVID-19. Early recognition of such changes may guide follow-up strategies in at-risk populations and improve our understanding of the long-term effects of this novel viral disease.

## Data Availability

No datasets were generated or analysed during the current study.
